# Risk alleles for IgA nephropathy-associated SNPs conferred completely opposite effects to idiopathic membranous nephropathy in Chinese Han

**DOI:** 10.1007/s12026-017-8947-6

**Published:** 2017-09-19

**Authors:** Xiaosong Qin, Chen Wang, Guanting Lu, Mengle Peng, Guixue Cheng, Hongquan Zhu, Yun Cao, Jianhua Liu, Yuzhong Li, Hong Cai, Funing Yang, Yanhong Liu, Xiaoyu Chen, Liubing Li, Nan Wan, Xiaoting Wen, Shijun Li, Ruili Nie, Dongchun Qin, Yongzhe Li, Yong Liu

**Affiliations:** 1 Department of Medical Laboratory, Shengjing Hospital of China Medical University, 36# Sanhao Street, Heping District, Shenyang, Liaoning 110004 China; 2 Graduate School of China Medical University, Liaoning, China; 3 Department of Rheumatology and Clinical Immunology, Peking Union Medical College Hospital, Chinese Academy of Medical Sciences & Peking Union Medical College, Key Laboratory of Rheumatology and Clinical Immunology, Ministry of Education, Beijing, China; 4 Department of Blood Transfusion, Tangdu Hospital, The Fourth Military Medical University, Shaanxi, China; 5 Department of Medical Laboratory, The First Affiliated Hospital of Zhengzhou University, Henan, China; 6 Department of Clinical Laboratory, The Second Hospital of Jilin University, Jilin, China; 7 Department of Laboratory Medicine, Yan’an People’s Hospital, Shaanxi, China; 8 Department of Laboratory Medicine, The Second Hospital of Dalian Medical University, Liaoning, China; 9 Department of Laboratory Medicine, The Second Hospital of Harbin Medical University, Heilongjiang, China; 10 Department of Laboratory Medicine, General Hospital of Shenyang Military Area Command, Liaoning, China; 11 Department of Laboratory Medicine, The First Affiliated Hospital of Dalian Medical University, Liaoning, China

**Keywords:** Immunoglobulin A nephropathy, Alleles

## Abstract

The coexistence of immunoglobulin A nephropathy (IgAN) and idiopathic membranous nephropathy (IMN) in a few cases suggested that there could be existed a similar mechanism in pathogenesis of these two types of primary glomerulonephritis. In order to verify this hypothesis, a total of 23 reported IgAN-associated SNPs were genotyped in a cohort of 485 IMN patients and 569 healthy controls with Chinese Han origin. After Cochran-Armitage test for trend analysis, seven IgAN-associated SNPs located in the major histocompatibility complex (MHC) region were found to be significantly associated with the susceptibility of IMN, with rs9275596 as the top one (*p* = 1.97E-43, OR = 3.977). It was worth mentioning that the minor alleles of the SNPs conferred completely opposite effects on the pathogenesis of IMN and IgAN, suggesting quite different roles played by these SNPs for these two kinds of primary glomerulonephritis. Conditional logistic regression analysis displayed that SNPs protective from IMN (odds ratio < 1.00) were still significantly associated with IMN (*p* = 3.67E-4 for rs660895 and *p* = 1.26E-4 for rs9275224) with the most significant SNP rs9275596 as a covariate. Haplotype-based analysis showed that the seven SNPs were mapped to independent linkage disequilibrium (LD) blocks. Moreover, three out of these seven SNPs, including rs9275224, rs660895, and rs9357155, were found to be potential expression quantitative trait loci (eQTLs) for HLA-DQ molecules. Out of the purpose of identifying the causal variants for IMN within the MHC region, imputation analysis was performed using genotype data of Chinese Han released by the 1000 Genome Project and identified hundreds of SNPs potentially associated with the disease. In brief, our analysis revealed a significant association with the susceptibility of idiopathic membranous nephropathy for the IgAN-correlated SNPs. These SNPs conferred a completely different role for the pathogenesis of these two kinds of diseases.

**Electronic supplementary material:**

The online version of this article (10.1007/s12026-017-8947-6) contains supplementary material, which is available to authorized users.

## Introduction

Idiopathic membranous nephropathy (IMN) and immunoglobulin A nephropathy (IgAN) are two common types of primary glomerulonephritis [1]. IMN is the most common type of nephritic syndrome primarily occurred in adults and is characterized by the immunoglobulin deposition in the glomerular capillary wall [2, 3]. As for IgAN, it is characterized by the positive IgA deposition in the meningeal region and is always found in pediatric population [4, 5].

Although IMN and IgAN are regarded as two distinct types of glomerular disease, cases of co-occurring IMN and IgAN have been reported previously in children and adults [6–9]. In our cohort of IMN samples, there are 4.78% (24/502) cases suffered from IgAN at the same time. The observation implied that there might be existed certain genetic factors associated with the pathogenesis of IMN and IgAN. It was also reported that genetic variants in genes of renin-angiotensin system (RAS), such as ACE (angiotensin I converting enzyme), AGT (angiotensinogen), and eNOS (endothelial nitric oxide synthase), were correlated to the development of IMN and IgAN [10].

In order to refine our understanding on molecular pathogenesis of IMN, this association study was conducted in a cohort of IMN patients and healthy controls with Chinese Han origin. The 23 SNPs that had been reported to be associated with IgAN by genome-wide association studies (GWASs) were genotyped to explore their association with the susceptibility of idiopathic membranous nephropathy. This study would shed light on the common genes or genetic polymorphisms implicated in the pathogenesis of both IMN and IgAN.

## Materials and methods

### Study populations

A total of 502 IMN subjects and 576 age-matched healthy controls were collected by Shengjing Hospital of China Medical University, Peking Union Medical College Hospital, and the First Affiliated Hospital of Zhengzhou University. The recruited cases and controls were unrelated individuals of Han Chinese ethnicity by self-report, and informed consent was obtained for each participant. The diagnosis of idiopathic membranous nephropathy was established by renal biopsy as part of the routine clinical workup for the investigation of proteinuria. Based on appropriate clinical and laboratory criteria, suspected secondary membranous nephropathy (SMN) subjects were excluded, consisting of malignancy and medications- associated MN, infection and toxins-associated MN, and autoimmune-associated MN. This study was approved by the ethics committee of Shengjing Hospital of China Medical University.

### Genotyping and quality control

Genomic DNA was extracted from peripheral blood of participants using QIAamp DNA mini kit (Qiagen, German). SNP genotyping was carried out by the MassArray iPLEX system (Agena, USA) at Beijing DNALead Co. LTD. All procedures were performed according to the manufacturer’s instructions. Ten nanograms of genomic DNA was amplified by multiplex PCR and then the amplicons were subjected to locus-specific single-base extension reactions. The extended products were desalted and transferred to a 384-element SpectroCHIP array. Allele detection was performed using MALDI-TOF mass spectrometry, and the MassArray TYPER software v4.0 (Agena, USA) was applied to analyze the mass spectrograms and assign genotype. Afterwards, we eliminated the subjects with call rate less than 80%, and the remaining subjects were further analyzed.

### Genotype imputation

The MaCH-Admix software was applied to impute the untyped SNPs within 32,000,000–33,200,000 on Chromosome (Chr) 6 [11]. The genotypes of 208 unrelated CHB subjects from the 1000 Genomes Project Integrated Phase 3 were applied as reference. The genotypes of cases and controls were imputed separately, and only the imputed SNPs with squared correlation between imputed and true genotypes (R-squared) ≥ 0.5 were further analyzed.

### Statistical test for association

PLINK tool set (http://pngu.mgh.harvard.edu/purcell/plink/) was applied to conduct the association analysis using the implemented Cochran-Armitage test for trend [12]. The odds ratio (OR) and 95% confidence interval (95% CI) were also calculated. The SNPs with *p* value less than 0.01 after Bonferroni correction for multiple testing were regarded to be significantly associated with IMN. The conditional logistic regression analysis was also performed using PLINK.

## Results

### Sample characteristics

Characteristics of the 485 qualified subjects with IMN and 569 controls were presented in Table 1. The cases and controls were age- and gender-matched. In our case group, the cases were predominantly male (66.1%), with a mean age at 48.1. As for the healthy control group, the gender ratio was 69.4%. There were 400 men and 176 women, with a mean age of 48.5 years. There were no significant differences with gender and age between case and control groups.

**Table 1:** Demographic and clinical features of subjects

Items	Cases	Controls	*p* value
Gender (Male / Female)	332 / 170	400 / 176	> 0.05
Ages range	14–84	20–89	
Mean ± SD	48.14 ± 14.05	48.57 ± 13.96	> 0.05
Routine test			
Serum creatinine (mmol/L)	80.27 ± 66.16		
Urea nitrogen (mg/dL)	6.41 ± 4.34		
Serum albumin (g/L)	28.01 ± 8.66		
Proteinuria (g/d)	4.83 ± 4.65		
eGFR (mL/min/1.73 m^2^)	97.54 ± 24.72		
Cholesterol (mmol/L)	6.17 ± 3.02		
Triglyceride (mmol/L)	3.72 ± 7.43		
Anti-PLA2R positive			
Not treated before assay	122 (68.92%)		
Treated before assay	70 (22.51%)		
Renal pathology			
Stage 1	86 (16.9%)		
Stage 1–2	65 (12.8%)		
Stage 2	302 (59.3%)		
Stage 2–3	25 (4.8%)		
Stage 3	30 (5.9%)		
Stage 3–4	2 (0.3%)		

*eGFR,* estimated glomerular filtration rate

### SNPs in MHC region were associated with IMN

For each of the recruited subject, a total of 23 SNPs that had been reported to be associated with IgAN were genotyped [13–15]. Association of 23 previously identified SNPs with the risks of IgAN in a Chinese population is shown in Table 2. After the elimination of subjects with call rate less than 80%, a total of 1054 samples, including 485 cases and 569 controls, were qualified for the subsequent association analysis. Cochran-Armitage test for trend was conducted for the 23 selected SNPs. As shown in Table 2, seven SNPs on Chr 6 were found to be significantly associated with IMN (*p* < 0.01 after Bonferroni correction), suggesting that these SNPs could be involved in the pathogenesis of both IMN and IgAN. All of these SNPs were located in the MHC region in the human genome. The risk alleles for rs9275596, rs2856717, rs7763262 rs9357155, and rs2071543 were higher in IgAN patients compared to controls while displaying lower in IMN. As for rs9275224 and rs660895, the frequencies of risk alleles were lower in IgAN compared to controls and higher in IMN. Most notably, for these SNPs, the risk alleles associated with IMN were found to confer a completely opposite effect in the pathogenesis of IgAN (Table 2). For example, the OR of the risk allele C for rs9275596 was 3.977 in IMN, compared with 0.31 in IgAN patients [13].

**Table 2:** Association of 23 SNPs with the risks of IMN in a Chinese population

SNP	Chr	Location (b37)	Gene	Risk allele	Allele frequency		*p* value (Bonferroni corrected)	Odds ratio (95% CI)
					Cases	Controls		
rs7763262	6	32424882	HLA-DQB1/HLA-DQA1	T	0.5237	0.3122	1.04E-20	2.423 (2.028–2.895)
rs660895	6	32577380	HLA-DRB1	G	0.05423	0.1843	3.57E-15	0.254 (0.184–0.351)
rs9275224	6	32659878	HLA-DQB1/HLA-DQA1	G	0.324	0.5655	1.74E-24	0.368 (0.308–0.441)
rs2856717	6	32670308	HLA-DQB1	A	0.5538	0.2465	4.64E-42	3.795 (3.153–4.567)
rs9275596	6	32681631	HLA-DQB1	C	0.4927	0.1963	1.97E-43	3.977 (3.278–4.825)
rs9357155	6	32809848	PSMB8	A	0.2707	0.1863	6.31E-05	1.621 (1.319–1.991)
rs2071543	6	32811629	PSMB8	T	0.2779	0.206	0.002058	1.483 (1.213–1.814)
rs1794275	6	32671248	HLA-DQB1	A	0.1054	0.1406	0.3745	0.720 (0.553–0.938)
rs2523946	6	29941943	HLA-A	C	0.4856	0.4621	0.2041	1.099 (0.925–1.304)
rs17019602	1	108188858	VAV3	G	0.2137	0.203	0.2103	1.067 (0.864–1.318)
rs3766404	1	196651832	CFH	C	0.07971	0.09315	0.1216	0.843 (0.620–1.146)
rs6677604	1	196686918	CFHR3-CFHR1 deletion	A	0.0701	0.07909	0.661	0.878 (0.633–1.218)
rs1883414	6	33086448	HLA-DPB2/HLA-DPB1	A	0.2427	0.2637	0.1784	0.895 (0.735–1.091)
rs3129269	6	33097614	HLA-DPB2	A	0.2215	0.2346	0.2016	0.929 (0.757–1.140)
rs10086568	8	6900336	DEFA	A	0.2696	0.268	0.6088	1.008 (0.831–1.223)
rs4077515	9	139266496	CARD9	T	0.3006	0.3222	0.2289	0.904 (0.751–1.088)
rs11150612	16	31357760	ITGAM/ITGAX	G	0.2593	0.2627	0.2139	0.982 (0.808–1.194)
rs11574637	16	31368874	ITGAM/ITGAX	C	0.003099	0.003515	0.09777	0.881 (0.197–3.948)
rs3803800	17	7462969	TNFSF13	A	0.2845	0.3222	0.06578	0.837 (0.694–1.009)
rs4227	17	7491177	MPDU1	G	0.1567	0.1907	0.05944	0.788 (0.628–0.990)
rs12537	22	30423460	MTMR3	T	0.2479	0.2206	0.2372	1.165 (0.951–1.426)
rs9983	22	30423744	MTMR3	A	0.09897	0.1046	0.3134	0.941 (0.708–1.249)
rs2412971	22	30494371	HORMAD2	A	0.3495	0.3515	0.3663	0.991 (0.828–1.186)

Next, we conducted conditional logistic regression analysis for the 23 SNPs. Once the most significant SNP rs9275596 was used as a covariate, the SNP rs660895 was still significantly associated with IMN (*p* = 0.0003671, Bonferroni corrected), and vice versa. Haplotype analysis showed that rs660895 was in high linkage disequilibrium (LD) with rs7763262 (D’ = 0.868 among all participants). In contrast, rs9275596 was mapped to a LD block constructed by rs9275224, rs2856717, and rs9275596. The frequency of haplotype AAC for this block was 0.197 and 0.494 in controls and cases, respectively (Table 4). Particularly, these three SNPs were also found to be the most significant SNPs associated with IMN and have the largest odds ratio (Table 3).

**Table 3:** SNPs significantly associated with IMN

SNP	Gene	Risk allele	Risk allele frequency for IMN		*p* value for IMN (Bonferroni corrected)	OR (95% CI) for IMN	OR for IgAN*
			Cases	Controls			
rs9275596	HLA-DR-DQ	C	0.493	0.196	1.97E-43	3.977 (3.278–4.825)	0.31
rs2856717	HLA-DR-DQ	T	0.554	0.247	4.64E-42	3.795 (3.153–4.567)	0.44
rs9275224	HLA-DR-DQ	G	0.324	0.566	1.74E-24	0.368 (0.308–0.441)	1.36
rs7763262	HLA-DR-DQ	T	0.524	0.312	1.04E-20	2.423 (2.028–2.895)	0.71
rs660895	HLA-DR-DQ	G	0.0542	0.184	3.57E-15	0.254 (0.184–0.351)	1.34
rs9357155	PSMB8	A	0.271	0.186	6.31E-05	1.621 (1.319–1.991)	0.35
rs2071543	PSMB8	T	0.278	0.206	0.00206	1.483 (1.213–1.814)	0.27

*The risk alleles for IgAN were obtained from previous association studies [13–15]

**Table 4:** Frequency of haplotype constructed by rs9275224, rs2856717, and rs9275596

Haplotype*	Frequency	
	Cases	Controls
AAC	0.494	0.197
GGT	0.323	0.559
AGT	0.124	0.195
AAT	0.059	0.050

*The haplotype frequency was calculated using HaploView software [16]

**Table 5:** RegulomeDB score for the significantly associated SNPs

SNP	Chr	Position (b37)	RegulomeDB score
rs9275224	6	32659877	1d
rs660895	6	32577379	1f
rs9357155	6	32809847	1f
rs2071543	6	32811628	4
rs7763262	6	32424881	6
rs2856717	6	32670307	6
rs9275596	6	32681630	6

1d, eQTL + TF binding + any motif + DNase peak

1f, eQTL + TF binding or DNase peak

4, TF binding + DNase peak

6, other

### Associated SNPs were mapped at expression quantitative trait loci (eQTLs)

Most of the seven SNPs significantly associated with IMN were mapped to non-protein coding regions in the human genome, except rs2071543 encoding a p.Gln49Lys non-synonymous amino acid in *PSMB8*. RegulomeDB is a database that annotates SNPs with known and predicted regulatory elements in the intergenic regions of the *Homo sapiens* genome. Known and predicted regulatory DNA elements include regions of DNase hypersensitivity, binding sites of transcription factors, and promoter regions that have been biochemically characterized to regulation transcription [16]. Therefore, we explored the RegulomeDB to investigate the function of these SNPs in gene expression regulation. As shown in Table 5, rs9275224, rs660895, and rs9357155 were found to be potential eQTLs according to RegulomeDB score, implying their role in gene expression regulation. Although the T/G polymorphism of rs2071543 encoded a missense variant, this SNP was reported to be a strong cis-eQTL for its association with increased expression of TAP2, PSMB8, and PSMB9 [17], which were involved in antigen processing and presentation.

### Genetic polymorphisms in MHC region was associated with IMN

In order to identify other SNPs associated with IMN, imputation was conducted for SNPs in Chr 6:32,000,000–33,200,000. After quality control (R-squared > 0.5), a total of 267 SNPs, including the seven SNPs significantly associated with IMN, were further analyzed. Regardless of the seven genotyped SNPs, 181 out of the 260 imputed SNPs were found to be significantly associated with IMN (Supplementary Table 1), suggesting that there might be quite a lot of genetic polymorphisms in MHC region associated with the pathogenesis of IMN. Among the imputed SNPs, the most significant signal for association with IMN was found at rs2858332, which was only 470 bp away from rs9275596 and in high LD with it (D’ = 1).

## Discussion

Idiopathic membranous nephropathy and IgA nephropathy are two distinct types of glomerular diseases. The former is characterized by the accumulation of immunoglobulin, complement components, and the M-type phospholipase A2 receptor (PLA2R1) in glomerular capillary wall, whereas the latter is characterized by IgA deposition in the meningeal region. In our cohort of IMN samples, 4.78% (24/502) cases were suffered from IgAN at the same time. This implied that there might be certain genetic factors associated with both of IMN and IgAN. Association analysis revealed that seven IgAN-associated SNPs in the MHC region were also significantly linked with IMN. The frequencies of risk alleles higher in IgAN patient compared to controls were lower in IMN and vice versa. Most notably, for these SNPs, the risk alleles associated with IMN were displayed to confer a completely opposite effect in the pathogenesis of IgAN (Table 2). This implied that, although MHC could be implicated in the pathogenesis of IgAN and IMN [18], the mechanism could potentially be quite different for these two types of primary glomerulonephritis.

Conditional analysis revealed that the seven SNPs might be located in two different LD blocks, which was proved by haplotype-based analysis. This result implied that even within the MHC region, the variants were associated with the pathogenesis of diseases through distinct mechanisms. Additionally, to investigate the roles of these SNPs in IMN pathogenesis, we surveyed the RegulomeDB, a database that annotates SNPs with known and predicted regulatory elements in the intergenic regions of the *Homo sapiens* genome [16]. At least three out of the seven SNPs were potential eQTLs to influence the expression of HLA-DQ molecules. Our analysis partially showed that the molecular mechanism of pathogenesis could be different between IgAN and IMN.

One of the crucial findings in this study was that, among the seven IgAN-associated SNPs in MHC region, the risk alleles significantly associated with higher IMN susceptibility were found to be lower in IgAN and vice versa. This result suggested a complex interplay between these two disorders. For these seven SNPs, the two alleles were associated with susceptibility of either IgAN or IMN. Actually, all of these SNPs were common SNPs with minor allele frequency ≥ 5% among healthy subjects. Therefore, it could be deduced that both IMN and IgAN were polygenic diseases, and the seven SNPs were merely associated with the susceptibility of IMN and IgAN, rather than causal mutations. We conducted epistasis for the seven SNPs to identify certain combination of genetic polymorphisms that might be significantly associated with IMN pathogenesis. However, no positive results were obtained (data not shown).

Overall, we identified seven IgAN-associated SNPs in the MHC region also significantly associated with IMN pathogenesis in Chinese Han. However, the alleles correlated with increased risk of IMN were found to be with decreased risk for IgAN, suggesting intertwined but different mechanisms for the pathogenesis of these diseases. These seven SNPs were potential eQTLs and might be involved in gene expression regulation. Furthermore, imputation analysis showed that hundreds of genetic polymorphisms in MHC region were potentially associated with IMN. Our analysis at least provided suggestive evidence for the similarity and difference in IMN and IgAN pathogenesis. The mechanism of these variants associated with the diseases should be extensively explored to reveal the clinical significance.

## Electronic supplementary material

Supplementary Table 1 (DOCX 35 kb)
