# Supplementary material for: Risk alleles for IgA nephropathy-associated SNPs conferred completely opposite effects to idiopathic membranous nephropathy in Chinese Han
Source: Immunol Res. 2017 Sep 19;65(5):1059–64. doi: 10.1007/s12026-017-8947-6 (PMC5613054; doi:10.1007/s12026-017-8947-6)
Supplement: Supplementary file 1 — (DOCX 35 kb) [file 12026_2017_8947_MOESM1_ESM.docx]

Supplementary Table 1. Imputed SNPs on Chr 6 significantly associated with IMN.

| SNP | Position (b37) | P value  (Bonferroni  -corrected) | Minor allele for IMN | Minor allele frequency | | OR  (95% confidence  interval) |
| --- | --- | --- | --- | --- | --- | --- |
|  |  |  |  | Cases | Controls |  |
| rs9268791 | 32421073 | 1.38E-19 | T | 0.5237 | 0.3128 | 2.415 (2.022-2.885) |
| rs9268803 | 32423237 | 1.38E-19 | A | 0.5237 | 0.3128 | 2.415 (2.022-2.885) |
| rs9268806 | 32423609 | 1.10E-17 | C | 0.5278 | 0.3295 | 2.275 (1.906-2.714) |
| rs9268808 | 32424176 | 8.92E-18 | G | 0.5299 | 0.3322 | 2.266 (1.9-2.704) |
| rs9268809 | 32424265 | 1.23E-17 | T | 0.5299 | 0.333 | 2.257 (1.892-2.693) |
| rs9268810 | 32424296 | 2.03E-17 | C | 0.5289 | 0.333 | 2.248 (1.885-2.682) |
| rs9268811 | 32424370 | 2.03E-17 | G | 0.5289 | 0.333 | 2.248 (1.885-2.682) |
| rs9268812 | 32424568 | 1.38E-19 | A | 0.5237 | 0.3128 | 2.415 (2.022-2.885) |
| rs9268814 | 32424616 | 2.54E-18 | C | 0.5278 | 0.326 | 2.311 (1.937-2.758) |
| rs9268815 | 32424677 | 1.38E-19 | A | 0.5237 | 0.3128 | 2.415 (2.022-2.885) |
| rs7743662 | 32425049 | 3.04E-18 | G | 0.5268 | 0.3251 | 2.311 (1.936-2.758) |
| rs7743415 | 32425135 | 1.38E-19 | C | 0.5237 | 0.3128 | 2.415 (2.022-2.885) |
| rs9268820 | 32425397 | 1.94E-18 | C | 0.5247 | 0.3207 | 2.338 (1.959-2.791) |
| rs7744304 | 32425487 | 1.38E-19 | G | 0.5237 | 0.3128 | 2.415 (2.022-2.885) |
| rs6457592 | 32425983 | 1.38E-19 | T | 0.5237 | 0.3128 | 2.415 (2.022-2.885) |
| rs7769693 | 32426458 | 5.24E-19 | T | 0.5237 | 0.3163 | 2.376 (1.99-2.838) |
| rs560530 | 32577222 | 2.61E-14 | A | 0.05155 | 0.1775 | 0.2518 (0.1824-0.3477) |
| rs661330 | 32577472 | 2.61E-14 | C | 0.05155 | 0.1775 | 0.2518 (0.1824-0.3477) |
| rs5002704 | 32659279 | 1.57E-22 | C | 0.3227 | 0.5589 | 0.376 (0.3146-0.4495) |
| rs5002705 | 32659319 | 3.62E-23 | T | 0.3196 | 0.558 | 0.3721 (0.3112-0.4448) |
| rs5002707 | 32659337 | 1.75E-22 | C | 0.3237 | 0.5598 | 0.3765 (0.315-0.4499) |
| rs5002708 | 32659357 | 1.75E-22 | C | 0.3237 | 0.5598 | 0.3765 (0.315-0.4499) |
| rs9275222 | 32659516 | 6.15E-20 | T | 0.299 | 0.5097 | 0.4103 (0.3427-0.4912) |
| rs4713587 | 32659535 | 1.57E-22 | T | 0.3227 | 0.5589 | 0.376 (0.3146-0.4495) |
| rs4248168 | 32659743 | 1.18E-22 | C | 0.3227 | 0.5598 | 0.3747 (0.3135-0.4479) |
| rs4713580 | 32659994 | 1.18E-22 | T | 0.3227 | 0.5598 | 0.3747 (0.3135-0.4479) |
| rs4713581 | 32660023 | 1.18E-22 | C | 0.3227 | 0.5598 | 0.3747 (0.3135-0.4479) |
| rs4713582 | 32660051 | 2.10E-22 | C | 0.3227 | 0.558 | 0.3774 (0.3157-0.4511) |
| rs4713583 | 32660153 | 1.18E-22 | G | 0.3227 | 0.5598 | 0.3747 (0.3135-0.4479) |
| rs4711304 | 32660170 | 2.80E-22 | C | 0.3227 | 0.5571 | 0.3787 (0.3169-0.4527) |
| rs4713584 | 32660237 | 1.18E-22 | T | 0.3227 | 0.5598 | 0.3747 (0.3135-0.4479) |
| rs9275225 | 32660262 | 1.18E-22 | A | 0.3227 | 0.5598 | 0.3747 (0.3135-0.4479) |
| rs9275226 | 32660311 | 2.10E-22 | T | 0.3227 | 0.558 | 0.3774 (0.3157-0.4511) |
| rs9275227 | 32660337 | 1.18E-22 | G | 0.3227 | 0.5598 | 0.3747 (0.3135-0.4479) |
| rs9275228 | 32660347 | 1.18E-22 | A | 0.3227 | 0.5598 | 0.3747 (0.3135-0.4479) |
| rs2858324 | 32660375 | 4.29E-43 | A | 0.5598 | 0.2452 | 3.915 (3.253-4.712) |
| rs9275230 | 32660442 | 2.80E-22 | G | 0.3227 | 0.5571 | 0.3787 (0.3169-0.4527) |
| rs9275231 | 32660505 | 1.57E-22 | C | 0.3227 | 0.5589 | 0.376 (0.3146-0.4495) |
| rs9275232 | 32660574 | 8.79E-23 | A | 0.3216 | 0.5589 | 0.3743 (0.3131-0.4474) |
| rs2647006 | 32660582 | 2.96E-43 | A | 0.5598 | 0.2452 | 3.915 (3.253-4.712) |
| rs9275234 | 32660586 | 1.18E-22 | T | 0.3227 | 0.5598 | 0.3747 (0.3135-0.4479) |
| rs9275235 | 32660587 | 1.18E-22 | G | 0.3227 | 0.5598 | 0.3747 (0.3135-0.4479) |
| rs9275236 | 32660601 | 1.18E-22 | G | 0.3227 | 0.5598 | 0.3747 (0.3135-0.4479) |
| rs9275237 | 32660612 | 1.17E-22 | A | 0.3216 | 0.558 | 0.3756 (0.3142-0.449) |
| rs9275238 | 32660689 | 1.18E-22 | G | 0.3227 | 0.5598 | 0.3747 (0.3135-0.4479) |
| rs9275239 | 32660717 | 1.18E-22 | A | 0.3227 | 0.5598 | 0.3747 (0.3135-0.4479) |
| rs9275240 | 32660722 | 1.18E-22 | T | 0.3227 | 0.5598 | 0.3747 (0.3135-0.4479) |
| rs9275241 | 32660741 | 1.18E-22 | T | 0.3227 | 0.5598 | 0.3747 (0.3135-0.4479) |
| rs9275242 | 32660774 | 1.18E-22 | T | 0.3227 | 0.5598 | 0.3747 (0.3135-0.4479) |
| rs9275243 | 32660784 | 1.57E-22 | G | 0.3227 | 0.5589 | 0.376 (0.3146-0.4495) |
| rs9275244 | 32660881 | 1.18E-22 | T | 0.3227 | 0.5598 | 0.3747 (0.3135-0.4479) |
| rs9275245 | 32660943 | 1.18E-22 | G | 0.3227 | 0.5598 | 0.3747 (0.3135-0.4479) |
| rs9275246 | 32661003 | 1.18E-22 | A | 0.3227 | 0.5598 | 0.3747 (0.3135-0.4479) |
| rs9275247 | 32661015 | 1.18E-22 | C | 0.3227 | 0.5598 | 0.3747 (0.3135-0.4479) |
| rs9275248 | 32661135 | 2.33E-22 | G | 0.3237 | 0.5589 | 0.3778 (0.3161-0.4515) |
| rs9275250 | 32661141 | 2.33E-22 | T | 0.3237 | 0.5589 | 0.3778 (0.3161-0.4515) |
| rs9275251 | 32661146 | 2.33E-22 | C | 0.3237 | 0.5589 | 0.3778 (0.3161-0.4515) |
| rs9275253 | 32661204 | 2.33E-22 | T | 0.3237 | 0.5589 | 0.3778 (0.3161-0.4515) |
| rs9275254 | 32661209 | 2.33E-22 | G | 0.3237 | 0.5589 | 0.3778 (0.3161-0.4515) |
| rs9275255 | 32661246 | 2.33E-22 | A | 0.3237 | 0.5589 | 0.3778 (0.3161-0.4515) |
| rs2858319 | 32661294 | 1.39E-41 | T | 0.5557 | 0.246 | 3.832 (3.185-4.611) |
| rs2858318 | 32661302 | 1.39E-41 | T | 0.5557 | 0.246 | 3.832 (3.185-4.611) |
| rs9275259 | 32661572 | 1.75E-22 | A | 0.3237 | 0.5598 | 0.3765 (0.315-0.4499) |
| rs9275260 | 32661575 | 1.75E-22 | T | 0.3237 | 0.5598 | 0.3765 (0.315-0.4499) |
| rs9275261 | 32661809 | 1.75E-22 | C | 0.3237 | 0.5598 | 0.3765 (0.315-0.4499) |
| rs2647011 | 32661915 | 3.10E-42 | C | 0.5577 | 0.246 | 3.864 (3.211-4.65) |
| rs375605509 | 32661933 | 2.87E-22 | C | 0.3165 | 0.5475 | 0.3828 (0.3201-0.4577) |
| rs73407347 | 32661960 | 1.75E-22 | T | 0.3237 | 0.5598 | 0.3765 (0.315-0.4499) |
| rs111391378 | 32662024 | 1.75E-22 | G | 0.3237 | 0.5598 | 0.3765 (0.315-0.4499) |
| rs72844346 | 32662033 | 7.21E-23 | G | 0.3206 | 0.5571 | 0.3752 (0.3138-0.4485) |
| rs72844347 | 32662059 | 1.75E-22 | G | 0.3237 | 0.5598 | 0.3765 (0.315-0.4499) |
| rs200419915 | 32662222 | 1.75E-22 | T | 0.3237 | 0.5598 | 0.3765 (0.315-0.4499) |
| rs201449386 | 32662239 | 1.75E-22 | C | 0.3237 | 0.5598 | 0.3765 (0.315-0.4499) |
| rs202170080 | 32662240 | 1.75E-22 | A | 0.3237 | 0.5598 | 0.3765 (0.315-0.4499) |
| 6:32662269:A:C | 32662269 | 1.57E-22 | C | 0.3227 | 0.5589 | 0.376 (0.3146-0.4495) |
| 6:32662270:G:A | 32662270 | 1.57E-22 | A | 0.3227 | 0.5589 | 0.376 (0.3146-0.4495) |
| rs2858317 | 32662280 | 3.03E-41 | C | 0.5536 | 0.246 | 3.8 (3.158-4.573) |
| rs9275263 | 32662344 | 1.09E-22 | G | 0.3237 | 0.5606 | 0.3751 (0.3139-0.4483) |
| rs9275264 | 32662372 | 1.75E-22 | G | 0.3237 | 0.5598 | 0.3765 (0.315-0.4499) |
| rs9275265 | 32662389 | 1.75E-22 | T | 0.3237 | 0.5598 | 0.3765 (0.315-0.4499) |
| rs9275266 | 32662392 | 1.75E-22 | C | 0.3237 | 0.5598 | 0.3765 (0.315-0.4499) |
| rs9275267 | 32662425 | 6.74E-23 | G | 0.3237 | 0.5615 | 0.3738 (0.3127-0.4468) |
| rs9275268 | 32662433 | 6.74E-23 | G | 0.3237 | 0.5615 | 0.3738 (0.3127-0.4468) |
| rs9275269 | 32662459 | 5.04E-23 | G | 0.3237 | 0.5624 | 0.3725 (0.3116-0.4452) |
| rs9275271 | 32662533 | 1.43E-23 | C | 0.3237 | 0.565 | 0.3685 (0.3083-0.4404) |
| rs9275272 | 32662546 | 8.38E-23 | C | 0.3175 | 0.5518 | 0.3778 (0.316-0.4518) |
| rs9275273 | 32662559 | 3.44E-23 | A | 0.3165 | 0.5527 | 0.3747 (0.3134-0.4481) |
| rs9275274 | 32662566 | 1.88E-23 | C | 0.3155 | 0.5527 | 0.3729 (0.3118-0.446) |
| rs9275276 | 32662676 | 1.43E-23 | C | 0.3237 | 0.565 | 0.3685 (0.3083-0.4404) |
| rs9275277 | 32662677 | 1.43E-23 | A | 0.3237 | 0.565 | 0.3685 (0.3083-0.4404) |
| rs9275279 | 32662843 | 2.32E-23 | A | 0.3237 | 0.5641 | 0.3698 (0.3094-0.442) |
| rs2856670 | 32662904 | 6.37E-41 | G | 0.5526 | 0.246 | 3.784 (3.145-4.553) |
| rs9275281 | 32662920 | 1.43E-23 | A | 0.3237 | 0.565 | 0.3685 (0.3083-0.4404) |
| rs9275282 | 32662974 | 2.86E-23 | T | 0.3247 | 0.5641 | 0.3715 (0.3109-0.4441) |
| rs2647022 | 32663004 | 6.37E-41 | A | 0.5526 | 0.246 | 3.784 (3.145-4.553) |
| rs9275284 | 32663073 | 2.86E-23 | T | 0.3247 | 0.5641 | 0.3715 (0.3109-0.4441) |
| rs9275285 | 32663080 | 2.86E-23 | G | 0.3247 | 0.5641 | 0.3715 (0.3109-0.4441) |
| rs9275286 | 32663143 | 1.76E-23 | C | 0.3247 | 0.565 | 0.3702 (0.3098-0.4425) |
| rs2647021 | 32663151 | 6.37E-41 | T | 0.5526 | 0.246 | 3.784 (3.145-4.553) |
| rs9275288 | 32663203 | 1.08E-23 | G | 0.3247 | 0.5659 | 0.3689 (0.3086-0.4409) |
| rs2647020 | 32663256 | 6.37E-41 | T | 0.5526 | 0.246 | 3.784 (3.145-4.553) |
| rs9275292 | 32663289 | 6.57E-24 | A | 0.3247 | 0.5668 | 0.3676 (0.3075-0.4394) |
| rs9275295 | 32663391 | 2.62E-23 | G | 0.3258 | 0.565 | 0.372 (0.3112-0.4445) |
| rs2647018 | 32663447 | 6.37E-41 | T | 0.5526 | 0.246 | 3.784 (3.145-4.553) |
| rs2856668 | 32663606 | 6.37E-41 | G | 0.5526 | 0.246 | 3.784 (3.145-4.553) |
| rs5000633 | 32663610 | 9.83E-24 | A | 0.3258 | 0.5668 | 0.3693 (0.309-0.4414) |
| rs2647016 | 32663925 | 6.37E-41 | C | 0.5526 | 0.246 | 3.784 (3.145-4.553) |
| rs2647013 | 32664284 | 6.37E-41 | C | 0.5526 | 0.246 | 3.784 (3.145-4.553) |
| rs2647012 | 32664458 | 6.37E-41 | T | 0.5526 | 0.246 | 3.784 (3.145-4.553) |
| rs2395522 | 32664722 | 2.23E-24 | T | 0.3268 | 0.5712 | 0.3645 (0.3049-0.4356) |
| rs2647003 | 32664880 | 6.37E-41 | T | 0.5526 | 0.246 | 3.784 (3.145-4.553) |
| rs2856667 | 32665079 | 1.06E-40 | T | 0.5598 | 0.2531 | 3.753 (3.122-4.512) |
| rs2647001 | 32665086 | 6.37E-41 | C | 0.5526 | 0.246 | 3.784 (3.145-4.553) |
| rs2856666 | 32665285 | 6.37E-41 | G | 0.5526 | 0.246 | 3.784 (3.145-4.553) |
| rs17206147 | 32665311 | 0.001823 | C | 0.04639 | 0.09842 | 0.4457 (0.3118-0.637) |
| rs2646998 | 32665367 | 6.37E-41 | T | 0.5526 | 0.246 | 3.784 (3.145-4.553) |
| rs2856727 | 32666397 | 6.37E-41 | C | 0.5526 | 0.246 | 3.784 (3.145-4.553) |
| rs2856725 | 32666738 | 6.37E-41 | C | 0.5526 | 0.246 | 3.784 (3.145-4.553) |
| rs2647040 | 32667280 | 6.37E-41 | A | 0.5526 | 0.246 | 3.784 (3.145-4.553) |
| rs2647042 | 32667548 | 6.41E-41 | C | 0.5588 | 0.2513 | 3.772 (3.137-4.536) |
| rs2647043 | 32667549 | 6.37E-41 | A | 0.5526 | 0.246 | 3.784 (3.145-4.553) |
| rs2856723 | 32667762 | 6.37E-41 | A | 0.5526 | 0.246 | 3.784 (3.145-4.553) |
| rs2856722 | 32667958 | 6.37E-41 | A | 0.5526 | 0.246 | 3.784 (3.145-4.553) |
| rs2858310 | 32668323 | 6.37E-41 | G | 0.5526 | 0.246 | 3.784 (3.145-4.553) |
| rs2647046 | 32668336 | 6.37E-41 | A | 0.5526 | 0.246 | 3.784 (3.145-4.553) |
| rs2647048 | 32668705 | 6.37E-41 | A | 0.5526 | 0.246 | 3.784 (3.145-4.553) |
| rs2858309 | 32668713 | 6.37E-41 | C | 0.5526 | 0.246 | 3.784 (3.145-4.553) |
| rs1612904 | 32669018 | 1.02E-39 | C | 0.4948 | 0.2039 | 3.825 (3.159-4.633) |
| rs9275389 | 32669132 | 6.37E-41 | C | 0.5526 | 0.246 | 3.784 (3.145-4.553) |
| rs79876547 | 32669137 | 0.001823 | C | 0.04639 | 0.09842 | 0.4457 (0.3118-0.637) |
| rs76901383 | 32669817 | 0.001823 | C | 0.04639 | 0.09842 | 0.4457 (0.3118-0.637) |
| rs3135001 | 32670136 | 8.46E-41 | T | 0.4546 | 0.1828 | 3.727 (3.063-4.536) |
| rs2856718 | 32670255 | 2.93E-24 | C | 0.6031 | 0.3691 | 2.598 (2.178-3.098) |
| rs35406540 | 32670368 | 0.001823 | G | 0.04639 | 0.09842 | 0.4457 (0.3118-0.637) |
| rs66709408 | 32670414 | 0.001823 | T | 0.04639 | 0.09842 | 0.4457 (0.3118-0.637) |
| rs2858305 | 32670464 | 6.37E-41 | G | 0.5526 | 0.246 | 3.784 (3.145-4.553) |
| rs35120848 | 32670495 | 0.001823 | T | 0.04639 | 0.09842 | 0.4457 (0.3118-0.637) |
| rs2647051 | 32670897 | 6.37E-41 | T | 0.5526 | 0.246 | 3.784 (3.145-4.553) |
| rs2856704 | 32671014 | 4.34E-24 | C | 0.6041 | 0.3717 | 2.579 (2.163-3.076) |
| rs2858304 | 32671044 | 4.34E-24 | G | 0.6041 | 0.3717 | 2.579 (2.163-3.076) |
| rs13192471 | 32671103 | 0.002898 | C | 0.04742 | 0.09842 | 0.4561 (0.3199-0.6501) |
| rs28451714 | 32671601 | 0.002898 | T | 0.04742 | 0.09842 | 0.4561 (0.3199-0.6501) |
| rs2856699 | 32671765 | 6.37E-41 | A | 0.5526 | 0.246 | 3.784 (3.145-4.553) |
| rs77184128 | 32672135 | 0.002898 | T | 0.04742 | 0.09842 | 0.4561 (0.3199-0.6501) |
| rs4568494 | 32672361 | 0.002898 | G | 0.04742 | 0.09842 | 0.4561 (0.3199-0.6501) |
| rs35030589 | 32672903 | 0.002898 | A | 0.04742 | 0.09842 | 0.4561 (0.3199-0.6501) |
| rs115580594 | 32673569 | 0.001823 | G | 0.04639 | 0.09842 | 0.4457 (0.3118-0.637) |
| rs9275511 | 32674329 | 7.33E-31 | G | 0.5866 | 0.3225 | 2.981 (2.494-3.563) |
| rs9275513 | 32674564 | 1.17E-35 | A | 0.5887 | 0.304 | 3.276 (2.737-3.921) |
| rs9275515 | 32674573 | 7.84E-36 | T | 0.5887 | 0.3032 | 3.289 (2.748-3.937) |
| rs9275516 | 32674643 | 1.60E-35 | A | 0.5887 | 0.304 | 3.276 (2.737-3.921) |
| rs9275517 | 32674649 | 1.60E-35 | A | 0.5887 | 0.304 | 3.276 (2.737-3.921) |
| rs9275518 | 32674700 | 1.24E-35 | G | 0.5835 | 0.297 | 3.316 (2.769-3.971) |
| 6:32674838:G:C | 32674838 | 6.37E-41 | G | 0.5526 | 0.246 | 3.784 (3.145-4.553) |
| 6:32674839:G:C | 32674839 | 4.76E-36 | G | 0.5897 | 0.3023 | 3.317 (2.771-3.971) |
| rs9275521 | 32674952 | 6.37E-41 | T | 0.5526 | 0.246 | 3.784 (3.145-4.553) |
| rs9275524 | 32675109 | 1.33E-34 | T | 0.5876 | 0.3058 | 3.235 (2.703-3.871) |
| rs9275526 | 32675237 | 6.62E-35 | G | 0.5876 | 0.3049 | 3.248 (2.714-3.888) |
| rs9275533 | 32675750 | 2.80E-37 | G | 0.5856 | 0.2926 | 3.416 (2.851-4.092) |
| rs9275535 | 32675789 | 4.22E-37 | G | 0.5856 | 0.2935 | 3.401 (2.839-4.074) |
| rs9275536 | 32675829 | 5.80E-37 | A | 0.5856 | 0.2935 | 3.401 (2.839-4.074) |
| 6:32675902:T:G | 32675902 | 1.77E-40 | T | 0.4948 | 0.2021 | 3.867 (3.192-4.685) |
| rs9275538 | 32676017 | 1.45E-38 | T | 0.5866 | 0.29 | 3.474 (2.899-4.163) |
| rs9275540 | 32676139 | 2.93E-39 | T | 0.5866 | 0.2873 | 3.519 (2.936-4.218) |
| rs3129721 | 32676251 | 2.99E-41 | C | 0.4948 | 0.2004 | 3.91 (3.226-4.738) |
| rs9275556 | 32677190 | 2.76E-39 | C | 0.5814 | 0.283 | 3.52 (2.936-4.221) |
| rs9275557 | 32677199 | 2.76E-39 | C | 0.5814 | 0.283 | 3.52 (2.936-4.221) |
| 6:32677669:C:T | 32677669 | 2.50E-39 | C | 0.5804 | 0.2812 | 3.536 (2.949-4.24) |
| rs9275565 | 32677938 | 3.24E-41 | T | 0.5804 | 0.2759 | 3.63 (3.026-4.355) |
| rs9275567 | 32678131 | 6.83E-41 | A | 0.5784 | 0.275 | 3.615 (3.013-4.338) |
| rs9275569 | 32678136 | 4.77E-41 | C | 0.5784 | 0.275 | 3.615 (3.013-4.338) |
| rs6932517 | 32678182 | 9.12E-42 | C | 0.5794 | 0.2733 | 3.663 (3.052-4.396) |
| rs9275570 | 32678199 | 3.41E-41 | G | 0.5794 | 0.2759 | 3.615 (3.013-4.337) |
| rs4947345 | 32678260 | 3.24E-44 | T | 0.5526 | 0.2373 | 3.97 (3.296-4.783) |
| rs3998157 | 32678477 | 5.29E-42 | C | 0.5784 | 0.2707 | 3.696 (3.079-4.437) |
| rs4273729 | 32678597 | 3.40E-42 | C | 0.5784 | 0.2698 | 3.713 (3.093-4.457) |
| rs9275572 | 32678999 | 8.97E-43 | A | 0.5784 | 0.2671 | 3.763 (3.134-4.519) |
| rs9275573 | 32679146 | 4.65E-44 | C | 0.5794 | 0.2636 | 3.848 (3.203-4.622) |
| rs3135461 | 32680122 | 7.75E-43 | G | 0.4948 | 0.1968 | 3.997 (3.296-4.847) |
| rs9275587 | 32680379 | 3.03E-44 | T | 0.5784 | 0.2627 | 3.849 (3.204-4.624) |
| rs2858332 | 32681161 | 5.26E-46 | G | 0.5784 | 0.2557 | 3.992 (3.32-4.8) |
